# Effects of psychological stress on the emission of volatile organic compounds from the skin

**DOI:** 10.1038/s41598-024-57967-2

**Published:** 2024-03-27

**Authors:** Géraldine Lucchi, Marine Crépin, Stéphanie Chambaron, Caroline Peltier, Laura Gilbert, Christelle Guéré, Katell Vié

**Affiliations:** 1 Centre des Sciences du Goût et de l’alimentation, CNRS, INRAE, Institut Agro, Université de Bourgogne, 21000 Dijon, France; 2 ChemoSens Facility, CNRS, INRAE, PROBE Research Infrastructure, 21000 Dijon, France; 3 Laboratoires Clarins, 5 rue Ampère, 95300 Pontoise, France

**Keywords:** Small molecules, Chemical biology, Psychology

## Abstract

Thirty-five women were included in a clinical study to characterize the volatile organic compounds (VOCs) emitted by the skin during exposure to psychological stress. An original silicon-based polymeric phase was used for VOC sampling on the forehead before and after stress induction. Cognitive stress was induced using specialized software that included a chronometer for semantic and arithmetic tasks. Assessment of stress was monitored using a State-trait anxiety inventory questionnaire, analysis of participants’ verbal expressions and clinical measurements. Identification and relative quantification of VOCs were performed by gas chromatography-mass spectrometry. Stress induction was validated by a significant increase in state-anxiety as indicated by the questionnaire, modifications in electrodermal activity measurements and the expression of stress verbatims. In parallel, a sebum production increase and a skin pH decrease were observed. A total of 198 VOCs with different potential sources were identified. They were categorized in 5 groups: probable cosmetic composition, VOCs produced by the body or its microbiota, environmental origin, and dietary intake. In our qualitative statistical approach, three VOCs were found to be correlated with stress induction and 14 compounds showed significance in the paired Wilcoxon test. Fatty-acyls derived from lipids were predominantly identified as well as ethylbenzenes.

## Introduction

The volatilome is the entire set of volatile organic compounds (VOCs) generated by a plant, a bacterial, or an animal organism^1^. The human volatilome consists of thousands of VOCs emitted through exhaled breath, saliva, blood, urine, milk, feces, and skin emanations^2^. These emanations are not exclusively associated with pathological phenomena; instead, they are released by all healthy human bodies^3^, resulting from chemical reactions occurring within the organs. Moreover, VOCs from the human body are not exclusively generated by chemical reactions occurring in the body, but they may also have an exogenous origin from environmental exposure and the use of products.

Endogenous skin volatiles are mainly produced by the eccrine, sebaceous, and apocrine glands interacting with naturally occurring bacteria on the skin's surface^4^. VOCs contribute to the olfactory profile of the skin, forming part of the odor print. Other factors, such as genetic characteristics (including the major histocompatibility complex), environment^5^, diet^6^, lifestyle^7^, use of cosmetic products^8^, diseases or metabolic disorders, and individual variations^9^, also contribute to the overall odor profile. According to a literature survey published in 2021, a compilation of 623 VOCs was identified in the skin volatilome of a healthy human body^10^.

The impact of psychological stress on the emission of VOCs from human skin is poorly documented. Stress is a cognitive process during which a subject evaluates and considers the threats and challenges of his environment. The endocrinologist Hans Selye introduced a theory in 1956, suggesting that stress is a reaction to an external stimulus^11^. Goodnite^12^ defined stress with three key points: firstly, as the application of tension, force, or pressure (a stimulus) to an organism; secondly as the appraisal of the stimulus as overwhelming, indicating that the organism perceives an inability to meet the challenge; and thirdly as a measurable response by the organism to the stimulus. Stress is not solely a stimulus or response but can be induced by the learning context (visual and/or sound interference) and by the interface used (temporal pressure and feedback)^13^. Stress-related emotional responses are accompanied by a set of physiological, cognitive^14^, and behavioral reactions. Physiological and physical responses are controlled by the vasomotor and autonomic nervous system and may include increased heart rate and blood pressure, sweating by the sweat glands, and elevated body temperature.

The cognitive assessment of stress is crucial because it enables the person to quantify the stressful event. The organism processes stimuli in a specific manner by evaluating them before triggering a particular emotional response. Various criteria are thus assessed, including the significance, novelty, and predictability of the event^15^. Studying the effects of stress on cognition and behavior involves designing and implementing stress induction protocols that ensure stress is manipulated as an independent variable, allowing for the establishment of influence and causal relationships within the research framework. The development of experiments on humans requires the creation of non-invasive procedures that guarantee limited effects, do not persist in the long term, and generate moderate levels of stress, sufficient to observe their effects without generating harm to the study participants. As mentioned by Ferreira^16^ “stress induction procedures must include elements of novelty, unpredictability, loss of control, representing a potential threat or loss, and, in some cases, social evaluation”. Only three publications have described the characterization of VOCs emitted during psychological stress in humans. In 2016, Martin et al. conducted a study on fifteen young adults^17^. Each volunteer participated in two sessions, one to induce a stress reaction (involving rapid resolution of arithmetic mental calculations) and the other not (exposed to soft music). The VOCs from the forehead area were sampled, and among them, four were modulated differently depending on the stress. In 2019, Tsukuda et al.^18^ carried out a study on thirty subjects on two separate days, inducing stress on the first day and a state of relaxation on the second day. The VOCs released in the armpits were sampled. They identified six stress biomarkers, different from those characterized by Martin et al. This discrepancy is not surprising, considering that the chemical nature of VOCs identified on the skin surface depends significantly on the sampling region due to variations in gland distribution across different body regions^19^. Finally, a study conducted in 2020 during the COVID-19 pandemic measured the stress levels in an academic student population^20^. For the stressful state, VOC samples were collected during a virtual exam from the forehead, while for the relaxation state, samples were gathered several weeks after the same students had completed the exam. Statistical analysis correctly classified the two states based on the VOCs profiles. However, in our opinion, the sampling between the relaxation and the stress phases should be as close as possible to avoid any external events that could artificially modify VOCs emissions. According to these authors, specific stress biomarkers do exist.

There are many different techniques available to characterize skin VOCs^21^. The sampling step is crucial, and the selection of the sampling device depends on the body area and the study's objective, whether it be prospective or clinical. Furthermore, the choice of the analytical method is closely related to the study's aim—identification, quantification, or profiling purposes^22,23^. Sensitivity and analysis throughput are also criteria to be considered. The sampling device must be physiologically safe, inexpensive, easy-to-use for non-laboratory operators, and single-use only, to be applied on a large number of volunteers and for safety requirements. A recently described polymer sorbent called Sorb-Star^®^, initially developed for forensic applications, fulfills all these criteria^24,25^.

Because psychological stress is known to induce skin barrier dysfunction and modulate the cutaneous inflammatory response^26^, this study focused on assessing the impact of psychological stress on the modulation of skin VOCs emission. The main objective of the study was to characterize the VOCs emitted by the skin on the forehead before and after the induction of psychological stress by the performance of simple cognitive tasks (solving operations, word scramble, etc.) in a group of middle-aged women (30–40 years), in order to identify the VOCs markers of psychological stress. The secondary objective was to assess the influence of skin surface conditions (pH, sebum, transepidermal water loss) on the VOCs emitted. Stress was induced by timed exercises on volunteers and validated through several physiological and clinical measurements. VOCs were collected on the forehead and analyzed using gas chromatography-mass spectrometry (GC–MS), the method of choice to separate and provide identification and quantification^27^. Finally, a qualitative and quantitative statistical approach was applied to all the results to discriminate the VOCs differentially expressed before and after the psychological stress tests. An attempt was made to classify these compounds according to their origin to better understand the mechanisms that lead to the emission of specific volatile compounds during psychological stress.

## Results

### Linearity, repeatability and stability of the sampling device

The linearity of the sampling devices was evaluated on the four VOC standards (isoamyl acetate, 2-phenylethanol, 2,3-dimethylpyrazine and heptanal). Calibration curves demonstrated linearity within the range of 5 to 25 ng/µL for each molecule. The R^2^ was greater than 0.99 (see Supplementary Fig. S1 online).

Repeatability of the method was assessed through three measurements conducted simultaneously within the linearity domain of the calibration curve, with the 15 ng/µL standard solution. The RSD were calculated for each volatile compound based on the mean relative abundances of two or three majority ions. The results obtained on day 0 ranged from 1.3 to 3.1% (see Supplementary Table S1 online). To assess the stability of the adsorbed molecules on the polymer, evaluations were carried out on days 3 and 12 after the sampling step to simulate the delay from invoicing to GC–MS analysis. On day 3, the RSD for each volatile standard at 15 ng/µL increased from 5.7% (2-phenylethanol) to 9.9% (heptanal). After 12 days of storage, RSD values were still under 10% for 2-phenylethanol, 2,3-dimethylpyrazine, and isoamyl acetate (3%, 4%, and 8.2% respectively), but exceeded 10% for heptanal (17.1%). Despite a slightly higher RSD after 3 and 12 days of storage, the average abundance of the two or three major ions of each standard was not drastically different and was in the same range.

### Optimization of the sampling procedure

Supplementary Fig. S2 online presents these results, without going into details. The conditioning step of the polymer facilitated the removal of impurities coming from the Sorb-Star^®^ itself. Thus, background noise was minimized to the maximum extent. Chromatographic profiles were relatively similar between static and dynamic sampling; however, peaks were more intense in the latter case, particularly after 15 min of sampling. No residues from the gloves were detected in the mass spectra after the GC–MS analysis. Lastly, although various VOCs were identified from the foaming gel used in the cleaning procedure, none of them were detected after desorption from the Sorb-Star^®^ when applied to the clean forehead.

### Validation of the stress induction

**Figure 1:**
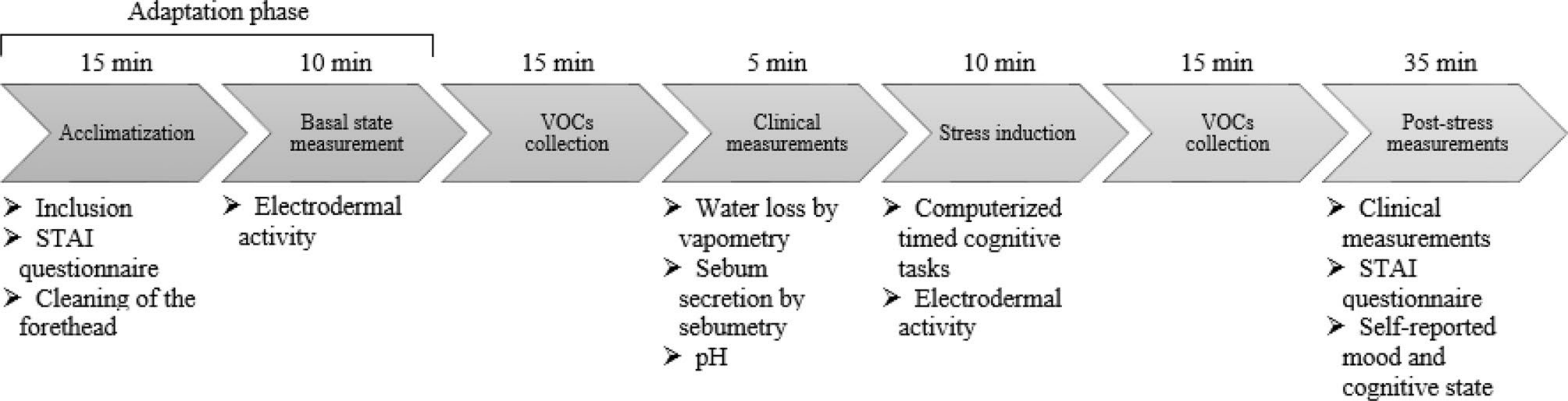
Procedure VOCs collection on volunteers: steps, measurements and time delay.

Figure 1 provides an overview of the various steps in the study. Table1 presents the results obtained from the State-Trait Anxiety Inventory (STAI) questionnaire and the electrodermal activity (EDA) measurements. There was no time limit for the questionnaires, but both scales were completed in less than 10 min. The trait-anxiety score significantly increased by 7.9% (from 38.0 to 41.0) and the state-anxiety increased significantly by 34.1% (from 32.2 to 43.3) between the “non-stressed” and the “stressed” phases.

**Table 1:** Mean values ± standard deviation of STAI and EDA parameters collected before and during stress.

Measure	Parameter	Before stress	During stress	p-value
STAI^1^	Trait-anxiety score	38.0 ± 8.6	41.0 ± 12.5	0.0356
	State-anxiety score	32.2 ± 8.2	43.2 ± 14.0	0.0001
EDA^2^	SCL^3^ (µS)	0.48 ± 0.31	1.9 ± 1.2	< 0.0001
	Frequency of NS-SCRs (peaks/min)	0.036 ± 0.08	4.01 ± 2.38	< 0.0001
	Amplitude of NS-SCRs (µS)	0.014 ± 0.009	0.063 ± 0.039	0.011

P-value was calculated from the Wilcoxon test performed on the data obtained before and during stress for each parameter.

*NS-SCRs* non-specific skin conductance responses.

^1^State-trait anxiety inventory, ^2^Electrodermal activity, ^3^Skin conductance level.

The EDA signal can be decomposed into two quantitative measures: Skin Conductance Level (SCL), related to the slow tonic shifts of EDA and skin conductance responses (SCRs), related to rapid phasic transient events. Non-specific SCRs (NS-SCRs) are the number of SCRs in a period of time produced after a sustained stimulus. Three parameters were extracted from the EDA signals recorded during the adaptation phase and the stress induction phase. The first parameter was the average SCL (µS). The second one was the frequency (peaks/min) and the third one was the average amplitude (µS) of the NS-SCRs. All these three parameters significantly increased during the stress phase. The mean value of SCL increased from 0.48 to 1.9 µS, while the frequency of NS-SCRs raised from 0.04 to 4.0 peaks/min and the amplitude of NS-SCRs varied from 0.01 to 0.06 µS.

**Figure 2:**
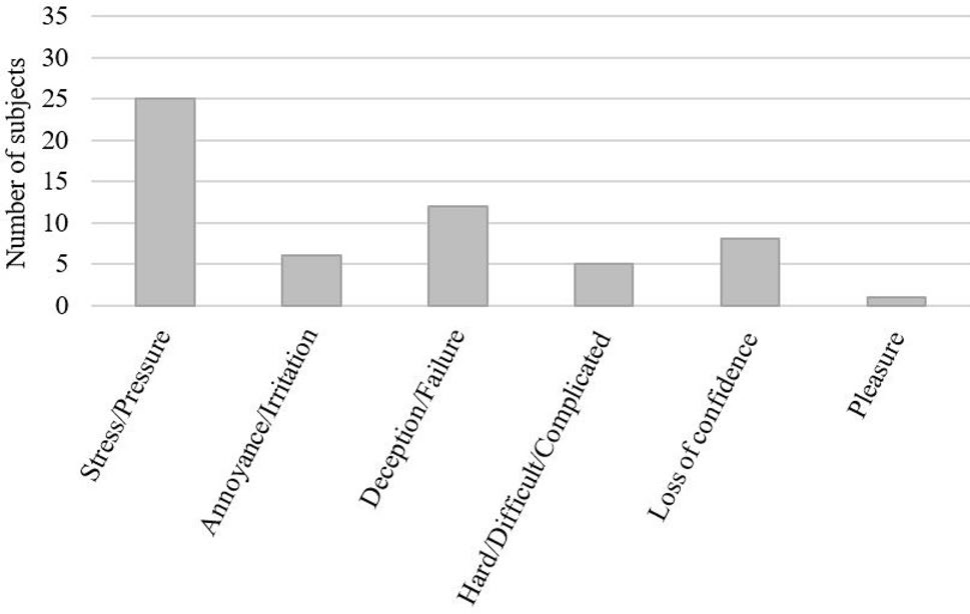
Self-reported mood and cognitive state of participants expressed at the end of the visit, in response to the open question “How did you feel during the tasks?”.

The participants' verbatims expressed throughout the study showed mainly the use of the lexical field of stress/pressure, deception/failure, hard/difficult/complicated for instance, by 71% of the participants (Fig. 2).

### Stress impact on basic skin parameters

**Figure 3:**
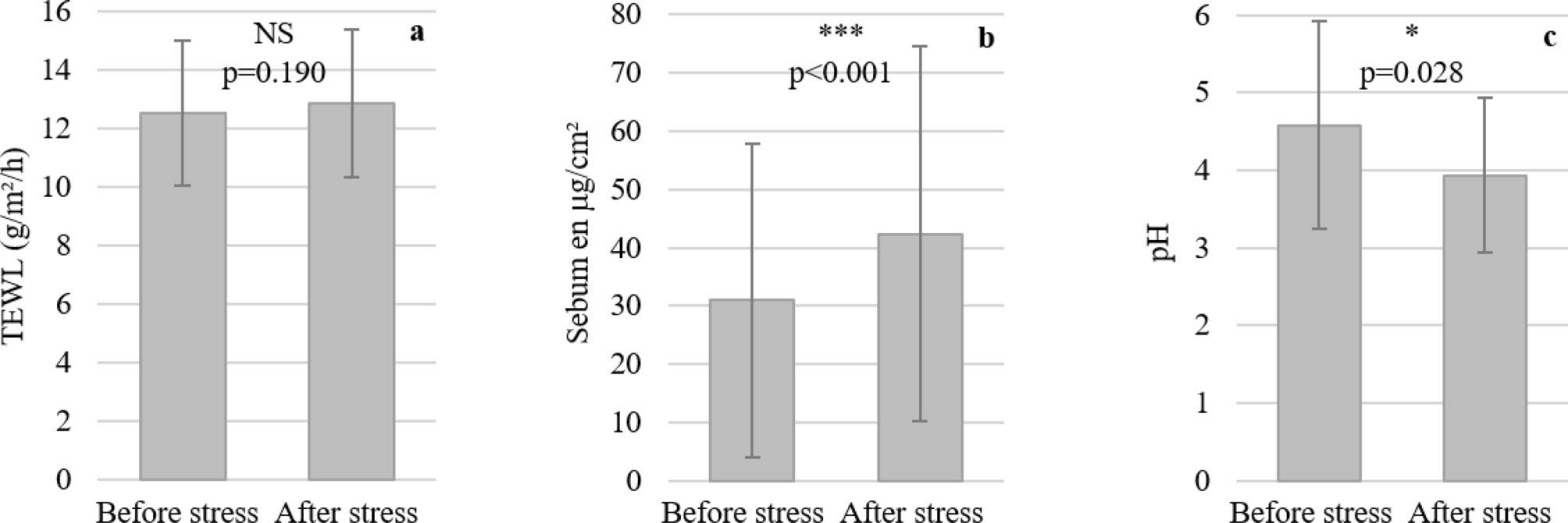
Measurements of Transepidermal Water Loss (TEWL) (**a**), sebum quantity (**b**) and pH (**c**) before and after stress phase. The significance level was α = 0.05. The normality of the data was assessed using a Shapiro–Wilk test. *NS* not significant; ***if p < 0.001; *if p < 0.05.

In this study, we compare basic skin parameters before and after a stressful situation. No significant difference was observed for the Transepidermal Water Loss (TEWL) measurement (Fig. 3a). We also measured the sebum presence in both stressed and neutral situations. Following the induction of psychological stress, the sebum level significantly increased by 37% on the subjects' foreheads (Fig. 3b). Simultaneously, we measured the cutaneous pH of the subjects, which significantly decreased by 14.1% after the stress situation (Fig. 3c).

### Identification and quantification of VOCs biomarkers linked to psychological stress

In terms of descriptive results, 198 VOCs were identified in this study (see Supplementary Table S2 online). All commonly found chemical classes were present: acids, aldehydes, halogen compounds, heterocyclic compounds and phenol were less represented, while alcohols, straight-chain alkanes, cyclic alkanes, esters, ketones and nitrogen compounds were more prevalent. These compounds were classified based on four possible origins (some compounds could originate from multiple sources). A total of 69 compounds were identified, likely originating from cosmetic compositions. Thirty-seven compounds could originate from human or microbiota metabolisms, while 33 originated from the environment. Numerous VOCs could originate from food (49 identified in this study). Finally, a few of them could not be classified due to their unknown origin.

For quantitative analyses (see Supplementary Table S3 online), the first eight samples (women 1 to 8) were excluded due to an issue with the GC autosampler (internal standards were not injected). Table 2 shows the three VOCs that were over-expressed (3-methylpentadecane; 2-hydroxyethyl acetate; 2-hydroxyethyl propanoate) with a significant stress effect in our qualitative approach (considering the presence or absence of VOCs in the samples).

**Table 2:** List of over-expressed VOCs significant with the qualitative approach.

Compounds names	CAS number	% of MV before stress	% of MV after stress	p-value (corrected with Bonferoni)	Concentration (ng/µL) after stress
3-methylpentadecane	2882–96-4	62.86	0	0.002	5.8
2-hydroxyethyl acetate	542–59-6	100	0	< 0.001	10.4
2-hydroxyethyl propanoate	24,567–27-9	100	2.86	< 0.001	3.6

The percent of missing values (MV) are reported before and after stress. This table is sorted by increasing p-value for the paired mac Nemar test. Concentrations are relative quantifications according to spiking standards.

Table 3 displays the 14 compounds that were over-expressed and significant for the paired Wilcoxon test. There were mostly part of the alkane family (2,6,10,14-tetramethylpentadecane, 2-methylpentadecane, 4-methylpentadecane, undecan-5-ylbenzene, 7-methylhexadecane, 2-methylhexadecane, 2,6,10-trimethylpentadecane, dodecan-6-ylbenzene, undecan-6-ylbenzene, 4-methylhexadecane, heptadecane). Additionally, one phenol (butylated hydroxytoluene), one ketone (geranyl acetone) and one nitrogen compound (N,N-dibutylformamide) were also characterized. The concentration calculation was performed by relative quantification using two standards spiked during SorbStar^®^ desorption. According to Tables 2 and 3, the concentration range strongly depended on the VOCs. The least represented compounds after the stress induction were quantified at 3.6 ng/µL (2-hydroxyethyl propanoate) while the two most abundant ones were geranyl acetone and butylated hydroxytoluene with 121.1 and 177.4 ng/µL, respectively.

**Table 3:** Mean values ± standard deviation (sd in brackets) of VOCs collected before and after stress.

Compounds names	CAS number	Average (sd) before stress	Average (sd) after stress	p-value (Wilcoxon)	Concentration (ng/µL) before stress	Concentration (ng/µL) after stress
2,6,10,14-tetramethylpentadecane	1921-70-6	5.85E + 06	8.67E + 06	2.57E-08	8.9	13.3
		(3.92E + 06)	(5.11E + 06)			
Butylated hydroxytoluene	128-37-0	2.48E + 07	1.44E + 08	3.67E-08	41.6	177.4
		(2.85E + 07)	(1.16E + 08)			
2-methylpentadecane	1560-93-6	2.40E + 06	4.41E + 06	1.17E-07	3.3	6.1
		(1.62E + 06)	(3.06E + 06)			
4-methylpentadecane	2801-87-8	1.81E + 06	2.67E + 06	3.15E-07	2.7	4.0
		(1.30E + 06)	(1.63E + 06)			
undecan-5-ylbenzene	4537-15-9	2.62E + 07	3.19E + 07	1.61E-06	40.4	51.5
		(2.04E + 07)	(2.26E + 07)			
7-methylhexadecane	26730-20-1	3.93E + 06	1.09E + 07	1.88E-06	7.0	16.0
		(2.52E + 06)	(7.71E + 06)			
2-methylhexadecane	1560-92-5	5.85E + 06	8.11E + 06	5.16E-05	8.7	12.3
		(3.19E + 06)	(3.90E + 06)			
2,6,10-trimethylpentadecane	3892-00-0	5.34E + 06	8.09E + 06	5.55E-05	6.5	11.4
		(4.33E + 06)	(5.85E + 06)			
dodecan-6-ylbenzene	2719-62-2	2.70E + 07	3.40E + 07	1.94E-04	36.9	43.1
		(2.18E + 07)	(2.46E + 07)			
Geranyl acetone	689-67-8	7.43E + 07	8.21E + 07	2.29E-04	104.3	121.1
		(5.07E + 07)	(5.44E + 07)			
undecan-6-ylbenzene	4537-14-8	2.76E + 07	3.43E + 07	2.52E-04	43.9	53.1
		(1.79E + 07)	(2.04E + 07)			
N,N-dibutylformamide	761-65-9	2.93E + 07	4.17E + 07	3.99E-04	41.1	47.9
		(2.69E + 07)	(5.23E + 07)			
4-methylhexadecane	25117-26-4	3.87E + 06	5.28E + 06	2.40E-04	4.8	7.0
		(2.64E + 06)	(3.33E + 06)			
Heptadecane	629-78-7	3.53E + 07	4.30E + 07	5.31E-03	58.8	74.7
		(2.24E + 07)	(2.30E + 07)			

P-value was calculated from the Wilcoxon test performed on the data obtained before and after stress for each parameter. Only significant VOCs were displayed. Concentrations are relative quantifications according to spiking standards.

## Discussion

Sorb-Star^®^ was previously validated on 80 COVs standards representative of the compounds likely to be present in human hand odor^25^. A recent publication also used Sorb-Star^®^ for a preliminary study to search for specific biomarkers for breast cancer, both after breast and hand sweat collection^28^. The skin cleaning step must be conducted meticulously, as reported in these publications, using an odorless soap and a careful rinsing procedure, to prevent contamination by exogenous VOCs and to selectively adsorb the VOCs emitted by the skin, without trapping sweat and sebum secretions. It is worth noting that sweat and sebum could be sources of skin VOCs. The cleaning process was carried out to establish the basal state of the skin. The validation of the sampling device and the sampling procedure were important before conducting this study. This allowed us to make relevant choices to successfully carry out our project. For example, the conditioning of the polymer was carried out systematically, even though this was not required by the supplier's specifications. The linearity of the sampling devices, important to perform relative quantification, indicated a direct relationship between the detection and the concentration range studied, underlining the excellent linearity of both the method and the equipment. The sampling with volunteers took place at the Clarins laboratories in Pontoise, while the analyses were conducted at the ChemoSens platform in Dijon, covering a distance of 360 km. Therefore, it was crucial to evaluate the stability and the repeatability of this polymer after several days of sampling. Thermodesorption is recognized for being challenging to control and can introduce variability into measurements. An RSD not exceeding 10% is considered acceptable, and in this case, repeatability was successfully validated with this adsorbent on days 0 and 3 after sampling. Despite not observing degradation of our standards in the polymer after 12 days of sampling, the RSD for heptanal was > 10%. Aldehydes are typically more susceptible to reactions than other chemical classes contained in our standard solution (a phenylalcohol, a pyrazine, and an ester) due to the lower electron density essential for molecular stabilization^29^. Therefore, for our experiments, it was strongly recommended to conduct VOC analysis as soon as possible after the sampling step to prevent a loss of repeatability. The other observations of these validation steps enabled us to establish a highly controlled sampling procedure with significant VOC intensity and without any external contaminations. Consequently, a fifteen-minute dynamic sampling, conducted with nitrile gloves after cleaning the forehead with fragrance-free soap from Bioderma^®^, was selected. We are aware that it is not entirely rigorous to extrapolate the results of our validation to all chemical classes found on the skin, given that we only tested an alcohol, a pyrazine, an ester, and an aldehyde in a solvent phase. However, the work of Cuzuel et al.^25^ validated the use of Sorb-Star^®^ on a large number of skin VOCs. Spiked standards prepared in a solvent (ethanol in our study) provided a good representation of direct sampling, as VOCs are deposited directly onto the sorbent phase. For us, rolling on the skin is closer to immersion because there is direct contact of the Sorb-Star^®^ with the skin. Another criticism can be made: indeed, the sampling duration (1 h versus 15 min) between the standard samples and the real samples were completely different. According to Action Europe, where the Sorb-Star^®^ was purchased, one to four hours of contact is necessary for the optimized transfer of molecules from the sample to the silicone. However, when we transitioned to sampling on our subjects, rolling the Sorb-Star^®^ on the forehead for an hour was impractical due to comfort, time constraints, and potential biases it might induce (such as COV emissions due to irritation, for example). Therefore, 15 min proved to be a suitable compromise, resulting in the identification of nearly 200 compounds.

Regarding the software we developed, we chose this type of stress inducer because it is non-invasive and non-traumatic. The selected cognitive tasks were kept simple enough to avoid cognitive overload. However, the semantic and arithmetic tasks chosen were not too easy in order to avoid a "ceiling effect" where everyone would easily succeed. If the tasks were too easy, participants might not be fully engaged (leading to low attentional engagement) and the stress induced by the time pressure of the chronometer would have less effect. The score range for the individual sub-tests of the STAI questionnaire is between 20 and 80, with higher values indicating greater anxiety. For the state anxiety scale, a cut-off score of 39–40 has been proposed to detect clinically significant symptoms^30^. Considering that the state-anxiety scale measures current feelings, it can be inferred that the conditions under which the timed cognitive tasks were performed represented a psychologically stressful situation for the subjects. EDA measures, which reflect autonomic innervation of sweat glands, have recently been used to assess sympathetic nervous system activation. All the parameters extracted from the EDA indicated a significant increase in physiological arousal between the two phases. Indeed, the average SCL shows a modulation of the basal level of intrinsic activity associated with psychological stress. The frequency and the average amplitude tend to increase when an emotionally stimulating event occurs^31–33^. In summary, all measured psychometric, physiological and behavioral parameters, such as verbatims, clearly indicate an increase in participants' stress during the performance of the time-limited cognitive tasks.

The impact of the stress on basic skin parameters was evaluated. No significant difference was observed for the TEWL measurement, indicating that the water evaporation in the skin was not impacted by psychological stress. Altemus et al.^34^ reported similar results, with no increase in TEWL on the forearm before psychological stress, but an increase in TEWL on the cheek. These varying results could be explained by the different measurement locations on the face. The skin is typically drier on the cheek than on the forehead, making any difference in TEWL more noticeable in this area. A prior study did not establish a link between psychological stress and sebum increase^35^. However, the two sebum measurements in that study were separated by at least two months, introducing the possibility of seasonal or other physiological changes in volunteers, which can be challenging to evaluate over such an extended period. In our study, both measurements were performed within an hour, making stress induction the sole modified factor. Additionally, in vitro studies on sebocytes indicate that treatment with corticotrophin releasing hormone, secreted during psychological stress periods, induces the lipogenic activity of sebocytes^36^, potentially increasing skin sebum production. Finally, a decreased cutaneous pH suggested that acidic molecules may have been secreted during the stress phase.

Most of the VOCs characterized in this study were not previously identified in the literature on skin VOCs. According to the bibliography, these compounds could have several possible origins. Previous studies on skin secretion detected many of them as cosmetic origin and revealed the persistence of these exogenous compounds after application^37^. A list of VOCs authorized in personal care products can be found, for instance, in Cosing, the European Commission database for information on cosmetic substances and ingredients^38^. These compounds could be used as fragrance (e.g., crotonic acid, nonadecane, tridecan-2-one, ethyl myristate), skin conditioning (e.g., 1-octoxyoctane, isoamyl laurate, tetradec-1-ene, 2,6-dimethylhept-5-enal), viscosity controlling (e.g., 2-butoxyethanol, dodec-1-ene, decane), solvent (e.g. eicosane, hexadec-1-ene, dibutyl adipate, 2,2,4,4,6,8,8-heptamethylnonane), skin emollient (e.g. undecan-1-ol, 2,6,10-dimethyldodecane, diisobutyl maleate) or preservative (e.g. 2-phenoxyethanol). Biochemical processes leading to VOC production from the body have not been well described, but some compounds identified in this study could originate from human or microbiota metabolisms. Alkanes (methylalkanes) and aldehydes (e.g. 3-methylbut-2-enal, heptanal) are produced from lipid peroxidation^39,40^. Alcohols (e.g. octan-1-ol, ethane-1,2-diol, 1-(2-hydroxypropoxy)propan-2-ol) have been frequently identified in skin secretion; they can be formed by the reduction of the respective acid or via pyruvate, citrate and glycolysis pathways^3^. Some ketones, such as 6-methylhept-5-en-2-one, are produced through oxidative degradation of squalene, the most abundant unsaturated compound in human sebum^41^. Surprisingly, the use of a few compounds identified in this study is prohibited, such as acrylonitrile, aniline, and acetamide. These compounds, along with 29 others, originate from the environment. The ethylbenzenes family (constituents of petrol) likely arises from the air pollution (e.g. undecan-5-ylbenzene, dodecan-6-ylbenzene, tridecan-6-ylbenzene)^3^ or from natural oily extracts used in daily life products (e.g., decan-5-ylbenzene, undecan-6-ylbenzene, dodecan-4-ylbenzene, undecan-2-ylbenzene)^42^. p-xylene is a result of exogenous exposure via household cleaning products, vehicle exhausts and cigarette smoke^17^. Chlorinated carbons (e.g. 2-chlorobuta-1,3-diene, 1-chlorohexadecane, 1-chloro-5-(1-chloroethenyl)cyclohexene) are widely used as refrigerants, propellants (in aerosol applications), adhesives and solvents. Nitrogen compounds (e.g. N,N-dibutylformamide, N,N-dibutylacetamide, dibutylamine) are part of the human exposome (aerosols, solvents, emulsifiers…). Finally, numerous VOCs can be found in raw food products. The Human Metabolome DataBase^43^ allowed us to identified some of them: 3-methylbut-2-enal (from tea leaf, red rice), (E)-2-methylpent-2-enal (onion, nuts), 4-methylheptadecane (pepper), 3-methyltetradecane (cereals), 3-methylheptadecane (vanilla), tetradec-3-ene (soybean).

Dunn et.al. proposed several well-known mechanisms in response to psychological stress, including the activation of several biological and biochemical pathways^44^. In our qualitative and quantitative approaches, we focused only on overexpressed molecules because it would be easier to test them in a future biocellular assay for validation. 3-methylpentadecane has never been described as a skin VOCs. This fatty acyl comes from lipid metabolism; it is also a natural product found in plants (peppercorns, vanilla) (PubChem CID: 17899). 2-hydroxyethyl acetate (ethylene glycol monoacetate) and 2-hydroxyethyl propanoate (ethylene glycol monopropionate) are part of the ethylene glycols family. Ethyl glycol acetate results from the reaction of the ethyl ether derived from monoethylene glycol (ethyl glycol) with acetic acid. This product is used in thinners and acrylic polymerization processes. Among the VOCs identified, only 86 had less than 50% of missing values and were selected for the quantitative approach (based on the intensities obtained for each VOC). Fatty-acyls derived from lipids were predominantly identified: heptadecane, 2-methylpentadecane, 4-methylpentadecane, 7-methylhexadecane, 2-methylhexadecane, 2,6,10-trimethylpentadecane and 4-methylhexadecane. Methylated hydrocarbons have been reported as endogenous products of oxidative stress. Numerous VOCs in this category have been identified as potential biomarkers of various cancers^45^. Heptadecane was identified as one of the stress biomarkers in the Tsukuda study^18^. 2-methylpentadecane was observed among six potential breath markers of stress in another pilot study, in response to the PASAT (Paced Auditory Serial Addition Test) intervention^46^. Geranyl acetone has often been described as skin VOCs. This compound has been mentioned as an oxidation product from the squalene precursor when ozone (a pollutant from the troposphere) reacted with this human skin lipid^38^. A high concentration of ozone leads to a drop in the natural antioxidants of the epidermis, produces reactive oxygen species, biomolecule oxidation, depletion of cellular antioxidant defenses, cell stress, cytotoxicity^47^ and thus weakens the natural skin barrier made up of lipids^48^. The skin can no longer fulfill its role as a shield against external aggressions, and positive associations between coarse wrinkles and ozone exceedances have been recently observed^49^. The ethylbenzenes (e.g. undecan-5-ylbenzene, dodecan-6-ylbenzene and undecan-6-ylbenzene) are well-known as constituents of petrol and likely arise from air pollution^3^ (although undecan-6-ylbenzene have been identified in natural oily extracts in plants). Another compound described as a pollutant is the N,N-dibutylformamide, released from aerosols and exhaust gases. 2,6,10,14-tetramethylpentadecane (pristane) and butylated hydroxytoluene (well-known as BHT) are used in cosmetics, as skin conditioning agents and as a substitute of toluene (antioxidant, fragrance) respectively^38^. These compounds are not products of human metabolism (as we assume according to the bibliography); they probably accumulate in the skin due to cosmetic or environmental exposures. It is known that psychological stress disrupts the epidermal permeability barrier^50,51^ and may have increased its excretion with the changes in the physiological properties of the skin observed in our study.

The use of internal standards allowed us to perform a relative quantification of our compounds. Wang et al.^52^ measured dynamically and in real-time the VOCs from breath and skin in a controlled chamber for a few molecular species to obtain a total emission rate per hour and per person. Among the 4 molecules used for the validation of the sample device, only isoamyl acetate and 2,3-dimethylpyrazine were retained for relative quantification because they were absent from the samples and did not co-elute with other compounds. The standards were injected into the liner containing the Sorb-Star^®^. As the desorption efficiency is never 100%, the peak area of each standard was plotted on the calibration curve obtained during the validation step. The concentration thus obtained was used for the relative quantification (compared to the standards) of the VOCs in each sample. We are aware of the approximation of this quantification, as the ionization response in mass spectrometry is very dependent on the physicochemical properties of the molecules. However, this method allowed us to determine an order of magnitude for each of the identified markers. This concentration will be very useful later to validate biologically the effect of these VOCs on skin explant cultures. Geranyl acetone was one of the most important stress biomarkers identified in our study. This is a relevant observation if we consider that this molecule comes from the oxidation of squalene, the most abundant unsaturated lipid in human sebum^8^. Finally, Frumin et al.^53^ studied human bodily secretions during handshaking and certain previous compounds were identified in the non-visible communication between humans. It could therefore be interesting to study chemosignaling communication^54^ in a stressed situation to evaluate the potential transmission of stress.

## Conclusion

VOCs profiling and biomarkers identification require robust sampling procedures and validation methods. To maximize the chance of detecting VOCs related to psychological stress, we developed a short protocol for VOCs collection, with less than one hour between the first phase (adaptation state measurement) and the second phase (stress induction). This minimizes the potential for artifacts. A series of clinical and physiological tests, as well as questionnaires, were used to validate the occurrence of stress in subjects. A large number of women were recruited to ensure sufficient statistical power. Although we originally aimed for 40 subjects, some of them had to be excluded from our study because they did not fulfill the inclusion criteria, which is always a critical point in a clinical study. Nevertheless, 35 subjects were selected for analysis. The originality of this project was based on the use of an adsorbent polymer that is not widely employed for VOCs sampling. However, Sorb-Star^®^ is not without interest for in vivo sampling, as it is single-use, perfectly safe, easy to handle, and inexpensive. In addition to these practical advantages, we conducted a strict evaluation of its ability, after sampling, to reflect the VOCs composition emitted by the skin. The background noise generated by the polymer itself was tested and did not lead to any overlap problems. The adsorption performances were also tested in terms of linearity, repeatability and stability, and were found to be totally satisfying. This polymer has also recently been successfully tested on food matrices in our laboratory (data not yet published). In order to identify stress biomarkers, statistical analysis was a major challenge. In the end, 17 molecules from different chemical classes, induced by cognitive stress were selected. The relative quantification of these compounds revealed a production range during the stress phase. They come from the fields of lipid metabolism, oxidative stress, air pollution and cosmetic applications. Some of them are already known as biomarkers for pathologies, but only one was described as stress-related. Future work will consider studying the potential effects of the modulation of these VOCs expression on skin physiology. Another perspective of this work could also involve studying their effects on human communication by understanding chemosignaling.

## Methods

### Chemicals and reagents

All standards were purchased from Sigma-Aldrich (Saint Quentin-Fallavier, France). Ethanol was purchased from Fisher Scientific (Loughborough, UK) and the fragrance-free soap (Atoderm Intensive Foaming Gel) from Bioderma^®^ (Lyon, France).

### Linearity, repeatability and stability of the sampling device

VOCs were collected using a silicon-based polymeric phase known as Sorb-Star^®^ and purchased from Action Europe (Sausheim, France). The dimensions of the polymer were a cylinder with a length of 20 mm and a diameter of 2 mm. The Sorb-Star^®^ allows for the exhaustive extraction of volatile compounds upon contact with the skin.

The Sorb-Star^®^ material was conditioned (35 °C for 0.2 min, then raised to 240 °C at 100 °C/min and held for 30 min) in a glass-liner through thermo-desorption (TDU, Gerstel, Mulheim and der Ruhr, Germany), to remove adsorbed impurities on the polymer. Subsequently, they were stored in an Agilent (Santa Clara, USA) inert amber glass vial, hermetically closed with a blue polypropylene cap and a red polytetrafluoroethylene seal, at room temperature.

Standards from four different chemical classes were selected to control adsorption performance. The compounds we chose belong to widely known chemical families, which were well-known in our laboratory, readily available in pure form for purchase, and easily detected in GC–MS. Additionally, we were also searching for molecules not typically found on the skin to serve as standards for relative quantification. A mixture of isoamyl acetate (ester), 2-phenylethanol (alcohol), 2,3-dimethylpyrazine (pyrazine) and heptanal (aldehyde) was prepared in ethanol, at a concentration of 5, 10, 15, 20 and 25 ng/µL. 1.5 mL of this mixture was introduced in a 10 mL glass vial containing the Sorb-Star^®^. The vial was then subjected to stirring for one hour at 130 rpm.

Linearity was assessed, and repeatability was tested within the linear range, using the 15 ng/µL solution. Stability over time was checked on days 0, 3, and 12 after the sampling step. All these conditions were evaluated in triplicates.

For data processing, the intensities of two or three major ions in the MS spectra were summed. An average and a standard deviation were calculated from the three repetitions. Residual standard deviation (RSD) was also determined by multiplying the ratio of the standard deviation to the average by 100.

### Optimization of the sampling procedure

The impact on the VOC profiles of various parameters was investigated to optimize the sampling procedure. A comparison between static and dynamic sampling methods was conducted. With the static mode, the Sorb-Star^®^ was directly placed on the clean forehead, covered with a sterile gauze and an adhesive. In the dynamic mode, the Sorb-Star^®^ was rolled across the entire forehead. The duration of the sampling step (5, 10 and 15 min) was investigated. The potential presence of contaminant peaks resulting from nitrile gloves was examined. Additionally, the VOC composition of the fragrance-free soap used during the skin cleaning protocol before the experiments was analyzed. 1.5 mL of foaming gel was added in a 10 mL glass vial. The Sorb-Star^®^ was immersed in the slimy liquid for one hour and subjected to agitation before the GC–MS analysis. Following the sampling procedure, each device was stored in an Agilent (Santa Clara, USA) inert amber glass vial, hermetically closed with a blue polypropylene cap and a red polytetrafluoroethylene seal.

### Panel

The study was conducted in accordance with the ethical principles of Good Clinical Practice and the Declaration of Helsinki. Our study received a favorable opinion from the ethics advisory committee (Centre Hospitalier Universitaire de Brest, France—IDRCB 2021-A00620-41). All participants included in this study provided written informed consent.

Thirty-five non-smoking women aged 24 to 40 (mean 34.9 ± 5.4 years) were recruited in the Clarins Laboratories in Pontoise, France. They were informed that they would be required to perform cognitive tasks, with no mention of stress specified before the conclusion of the visit. Volunteers were asked not to consume coffee, mint, spices, fried food, garlic, or onion, and were advised not to engage in intense exercise for 24 h before the session. The use of essential oils, cosmetics, perfume, and make-up was also prohibited. Additionally, 24 h before the experiments, participants were instructed to exclusively use the fragrance-free soap Atoderm Intensive Foaming Gel from Bioderma.

### Stress induction

In contrast to the studies by Tsukuda et al.^18^ and Acevedo et al.^20^, our samples were collected on the same day, within a 2-h appointment, starting with an adaptation phase to evaluate the basal state of each subject. Figure 1 provides an overview of the various steps in the study. Once they arrive at the laboratory, participants are welcomed and given about 20 min to make themselves comfortable before starting the study. After this acclimatization period, inclusion questions were asked to ensure adherence to instructions.

The initial phase of the experiment involved the non-stressed condition. Women completed a State-Trait Anxiety Inventory (STAI) questionnaire^55^. Their forehead was cleaned with a fragrance-free soap by an operator wearing nitrile gloves, then rinsed carefully with clear water and dried with a paper towel. This step aimed to standardize the skin condition of all volunteers before collecting VOC samples. Electrodermal activity (EDA) (Shimmer3 GSR + , Shimmer, MA, USA) was then measured during 10 min, corresponding to the basal state measurement. Subsequently, the SorbStar^®^ was rolled over the entire surface of the subject's forehead for 15 min to collect the first VOC sample. As a control, a polymer was placed in the room where the sampling took place for 15 min to collect the exogenous VOCs present in the air during the study. Clinical measurements, including transepidermal water loss, sebum secretion, and cutaneous pH, were also recorded.

The second step of the experiment was the stress induction phase. Cognitive stress was introduced to the subjects through the completion of four different cognitive tasks conducted on a computer. The presence of a chronometer, imposing temporal constraint on the execution times of various simple arithmetic and semantic cognitive tasks, served as the stress inducer. This stress induction poses no risk to the participants.

A computer program (in .exe format) was specifically developed for the study by the computer science department of the Centre des Sciences du Goût et de l’Alimentation – CSGA. It included a sequence of four timed tasks: (1) a mental arithmetic task; (2) counting by steps of “13" from 0 to 520 task; (3) a word scramble task; (4) a word grid task. Each task had to be completed within a limited time (ranging from 1′30 min and 2 min depending on the task). The time course was displayed on the screen, with the remaining time shown in red during the last 20 s. In addition, a beep was emitted intermittently during the last 20 s of each task. Once the chronometer started, it was not possible to stop it or interrupt the exercise sequence. The four tasks were performed in a random order defined by the computer program.

During the stress phase, the EDA was recorded to evaluate differences between the basal state and stress phases. A second VOCs collection was completed immediately after the cognitive tasks, along with all clinical measurements, and a second administration of the STAI questionnaire. After each volunteer session, the VOCs samples were stored at 4 °C before being sent once a week, to the ChemoSens Platform of the CSGA in Dijon for analysis.

### Stress evaluation

A standardized questionnaire, Spielberger's STAI, one of the most widely used self-assessment scales for anxiety, was used for the independent quantification of current anxiety at the time of administration (state-anxiety part) and the subject's usual anxious temperament (trait-anxiety part). According to Ansseau^56^, it consists of two distinct parts, evaluating trait-anxiety and state-anxiety independently, each containing 20 items graded into four levels based on intensity or frequency. The trait-anxiety scale primarily assesses anxious personality characteristics (general feelings questionnaire), while the state-anxiety scale measures changes induced by various experimental situations (current feelings questionnaire). The participants completed this questionnaire themselves at the beginning and at the end of the experiment after all measurements were taken. Each item of the trait-state anxiety inventory was scored from 1 to 4, depending on its intensity concerning state anxiety (no = 1, rather no = 2, rather yes = 3, yes = 4) and its frequency concerning trait-anxiety (almost never = 1, sometimes = 2, often = 3, almost always = 4). For items indicating the absence of anxiety (19 out of the total 40), scoring was reversed. The scores for each item were summed, resulting in a total score ranging from 20 to 80 for each scale.

An EDA measurement was also conducted to evaluate the changes in the electrical conductance of the skin, strongly correlated with sweat production. The physiological components of the EDA were analyzed with the Imotions Software v.9.0 (iMotions, MA, USA). Two dry Ag/AgCl electrodes were positioned on the palm with Velcro strips at the medial phalanges of the index and middle fingers of the non-dominant hand of the subjects. The signal was collected at a sampling rate of 10 Hz, and a low-pass filter of 5 Hz was applied to smooth the signal. Finally, a high-pass filter of 0.05 Hz was used to retain the phasic component.

Finally, participants' spontaneous verbatims throughout the study were recorded, and the frequency of quotations was analyzed. At the end of the study, participants were asked to describe how they felt during the exercises, particularly the stress induction task.

### Skin clinical measurements

The clinical measures, including pH, sebum, Transepidermal Water Loss (TEWL) were conducted to evaluate the impact of stress on basic skin parameters. These evaluations were performed both before and after inducing stress to facilitate a comparison of measurements in both scenarios. All assessments were carried out on the temples of the subjects to avoid interference with the collection of VOCs from the forehead. Three independent measures were taken for each parameter. TEWL measurements were obtained using a Vapometer (Delphin Technologies, Kuopio, Finland); the evaporation rate was calculated in g/m^2^/h. Sebum secretion was monitored using a sebumeter (Courage & Khazaka, Köln, Germany). The sebum content was expressed in µg/cm^2^. Cutaneous pH was measured by a Skin pHmeter (Courage&Khazaka, Köln, Germany).

### VOCs analysis

Background noise generated by the Sorb-Star^®^ itself was evaluated before and after the conditioning step. The same protocol was applied for both the method validation and the analytical process. VOCs were desorbed from the Sorb-Star^®^ using the thermal desorption unit (TDU, Gerstel, Mulheim and der Ruhr, Germany) of a 7890A gas chromatography apparatus (Agilent Technologies, Palo Alto, USA) under the following conditions: initial temperature at 30 °C for 0.2 min, until 240 °C at 100 °C/min and held to 5 min. The desorbed analytes were cryo-focused at − 50 °C using liquid nitrogen in a Cool Injection System injector (CIS 4, Gerstel, Mulheim and der Ruhr, Germany). The compounds were then transferred into the GC–MS instrument according to the programmed temperature as follows: final temperature of 240 °C at 12 °C/s (held for 5 min). For relative quantification, only isoamyl acetate and 2,3-dimethylpyrazine were retained from our four standards, as 2-phenylethanol and heptanal co-eluted with VOCs from the skin. A mixture of 1µL of isoamyl acetate and 2,3-dimethylpyrazine at a concentration of 50 ng/µL was injected using the glass syringe of the Gerstel auto sampler into the liner of the apparatus. Helium was used as the carrier gas to separate volatile compounds, at a velocity rate of 40 cm/s, on a MEGA-WAX column (30 m × 0.25 mm i.d, 0.5 µm film thickness; MEGA, Legnano, Italy). Chromatographic separation conditions were programmed from 40 to 240 °C at 5 °C/min and held to 5 min. The transfer line temperature was set at 240 °C.

Mass spectrometry analyses were conducted using a 5975C mass selective detector (MSD, Agilent Technologies, Palo Alto, USA) in electron impact mode at 70 eV, with a scan rate of 4 scans/s. The analysis covered a mass-to-charge ratio range from 29 to 350 Da, with a source temperature set at 230 °C and a detector temperature at 150 °C. After subtraction of the background noise (chromatogram from Sorb-Star^®^ after air sampling in the room and silicone derivative peaks), molecules were identified by comparison of the experimental linear retention index and the obtained spectra, thanks to several databases, including the US National Institute of Standards and Technology (NIST08), WILEY11N17, and in-house INRAMass databases. Data processing was performed using the MSD ChemStation software. Relative quantification was based on the mean peak areas of the standards compared to those of each VOCs present in the samples.

### Statistical data analyses

All clinical skin and stress evaluation data (STAI, EDA) were analyzed using XLSTAT 2021.3.1 (Addinsoft, Paris, France) with a significance level of α = 0.05. The normality of the data was assessed using a Shapiro–Wilk test. Since the data did not follow a normal distribution, non-parametric two-tailed Wilcoxon signed-rank tests were performed on all the parameters to highlight differences between both the “non-stressed" and "stressed "phases.

Two approaches were developed in order to find volatile markers of stress. The first one was qualitative and consisted in considering the presence or absence of VOCs in the samples. Therefore, the data was encoded as presence or absence. A MacNemar test (equivalent to a paired Chi-Square test)^57^ was conducted in order to test whether the stress impacted the absence/presence of each VOC. The second one was quantitative and based on the intensities obtained for each VOC. A paired Wilcoxon test was conducted in order to test whether the observed intensities were significantly higher (or lower) after the stress period^58^. This test was exclusively applied to VOCs with less than 50% of missing values. In both approaches, the resulting p-values were corrected with Bonferroni.

Finally, the Spearman correlation coefficients, along with their associated significance tests, were computed to assess the correlation between the differences before/after stress in identified potential chemical markers of stress and clinical variables. The statistical analysis was carried out using R (version 4.0.2). The macNemar test was computed with the ‘stat’ packages. The R code used for the analysis is available on www.github.com/ChemoSens/ExternalCode/SkinAnalysis.

### Supplementary Information

Supplementary Information.

Supplementary Table S3.

## Data Availability

The datasets used for STAI questionnaires, electrodermal activity and skin parameters are available from the corresponding author. All data generated after GC–MS analysis (raw data) during this study are included in the supplementary information files. All authors of this manuscript give their consent to share this data.
