# Supplementary material for: Effects of psychological stress on the emission of volatile organic compounds from the skin
Source: Sci Rep. 2024 Mar 27;14:7238. doi: 10.1038/s41598-024-57967-2 (PMC10973438; doi:10.1038/s41598-024-57967-2)
Supplement: Supplementary file 1 — Supplementary Information. [file 41598_2024_57967_MOESM1_ESM.docx]

**Supplementary information**


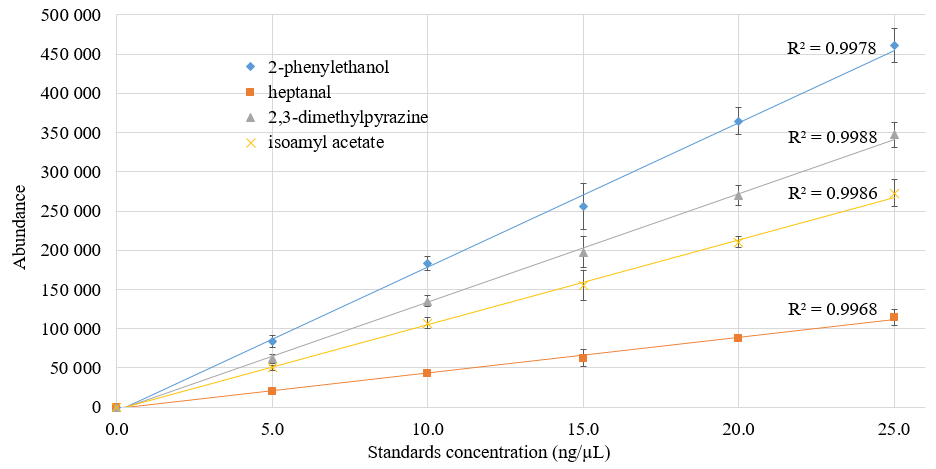


**Supplementary Figure S1.** Calibration curves representing the abundance of the retained major ions of the four standard compounds according to their concentration (ng/µL). The data were obtained by GC-MS with the thermal desorption unit. For each curve, the coefficient of determination (R^2^) are displayed.

| Standards | 2-phenylethanol | heptanal | 2,3-dimethylpyrazine | isoamyl acetate |
| --- | --- | --- | --- | --- |
| Day 0 | | | | |
| MRA Sorb-Star^®^ 1 | 130,696 | 51,061 | 329,204 | 339,023 |
| MRA Sorb-Star^®^ 2 | 129,499 | 52,557 | 334,225 | 343,938 |
| MRA Sorb-Star^®^ 3 | 132,916 | 53,617 | 320,470 | 323,796 |
| Average | 131,037 | 52,412 | 327,966 | 335,586 |
| Standard deviation | 1,734 | 1,284 | 6,961 | 10,502 |
| RSD % | 1.3 | 2.5 | 2.1 | 3.1 |
| Day 3 | | | | |
| MRA Sorb-Star^®^ 4 | 133,942 | 60,403 | 381,640 | 409,249 |
| MRA Sorb-Star^®^ 5 | 119,912 | 53,087 | 352,653 | 367,649 |
| MRA Sorb-Star^®^ 6 | 124,080 | 49,911 | 333,843 | 355,607 |
| Average | 12,978 | 54,467 | 356,045 | 377,502 |
| Standard deviation | 7,205 | 5,380 | 24,078 | 28,146 |
| RSD % | 5.7 | 9.9 | 6.8 | 7.5 |
| Day 12 | | | | |
| MRA Sorb-Star^®^ 7 | 151,346 | 67,259 | 438,997 | 502,020 |
| MRA Sorb-Star^®^ 8 | 145,528 | 49,529 | 422,093 | 493,897 |
| MRA Sorb-Star^®^ 9 | 142,638 | 51,844 | 405,252 | 431,104 |
| Average | 146,504 | 56,211 | 422,114 | 475,674 |
| Standard deviation | 4,435 | 9,638 | 16,873 | 38,812 |
| RSD % | 3.0 | 17.1 | 4.0 | 8.2 |

**Supplementary Table S1.** Repeatability and stability of the Sorb-Star^®^ were assessed at 0, 3, and 12 days after sampling using four compounds as standard solution. The Relative Standard Deviation (RSD) was calculated based on the mean relative abundances (MRA) of two or three major ions at 0, 3, and 12 days after the sampling step.


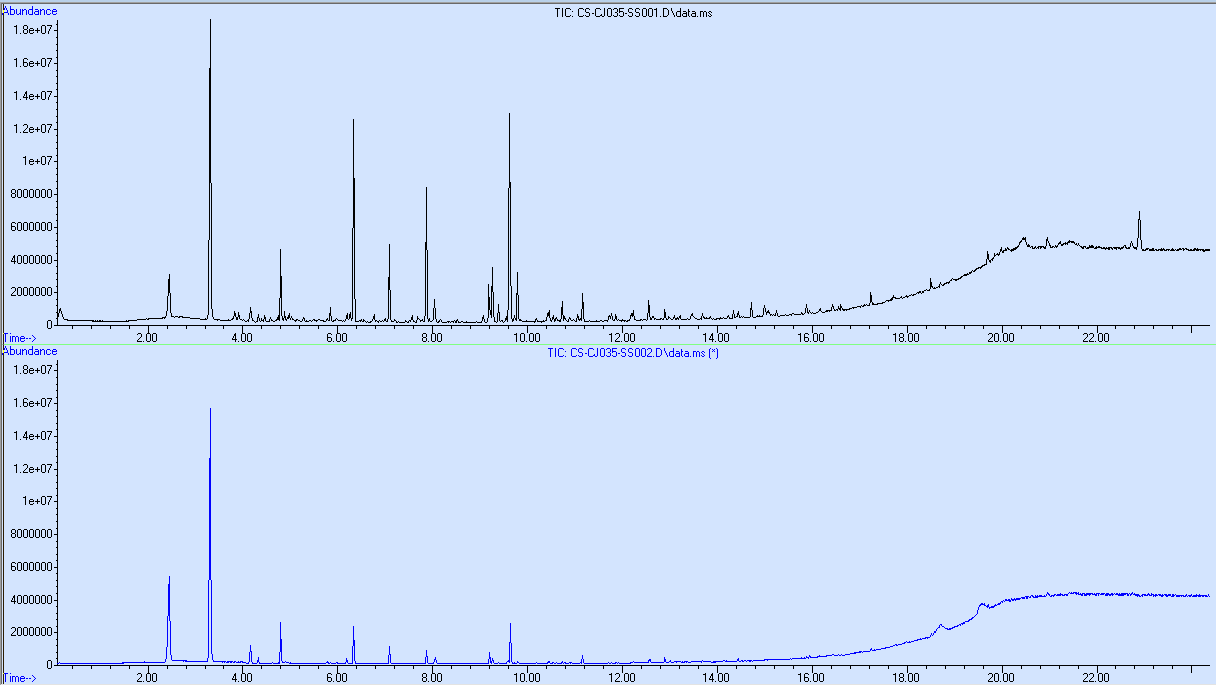


b

a


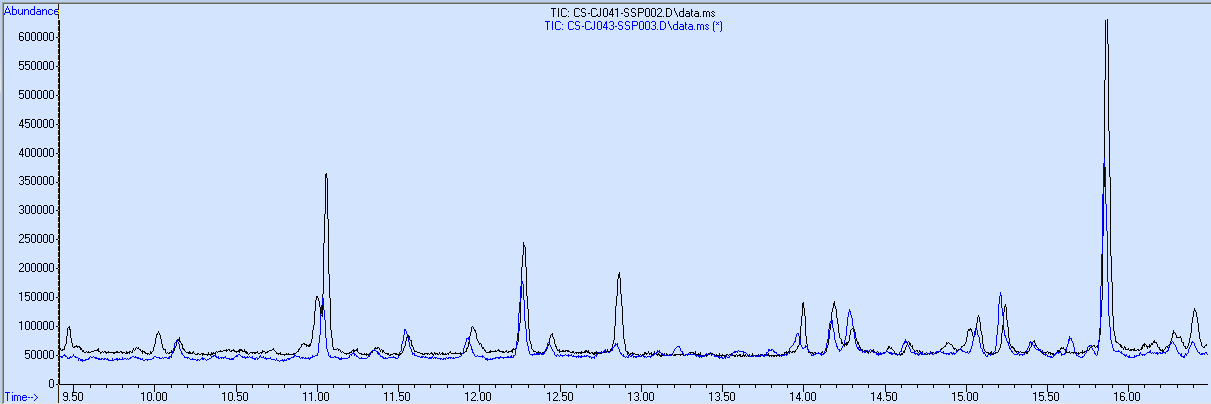


c


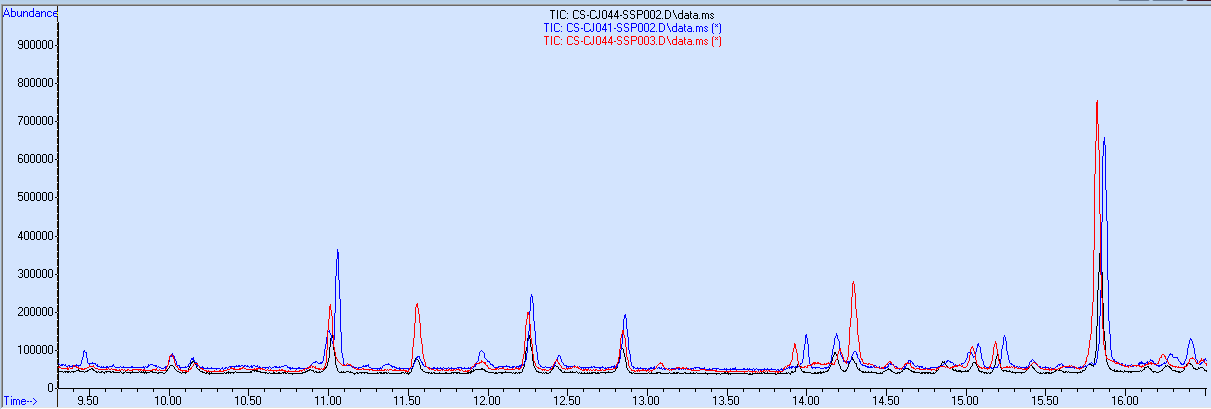


d


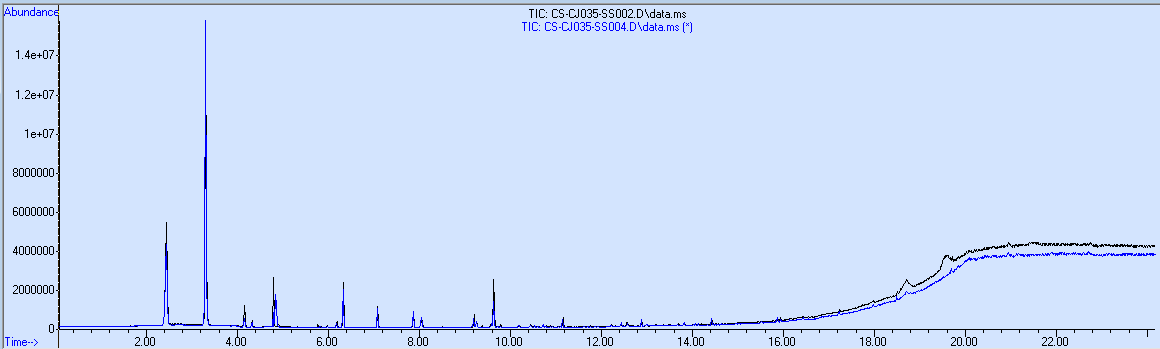


e


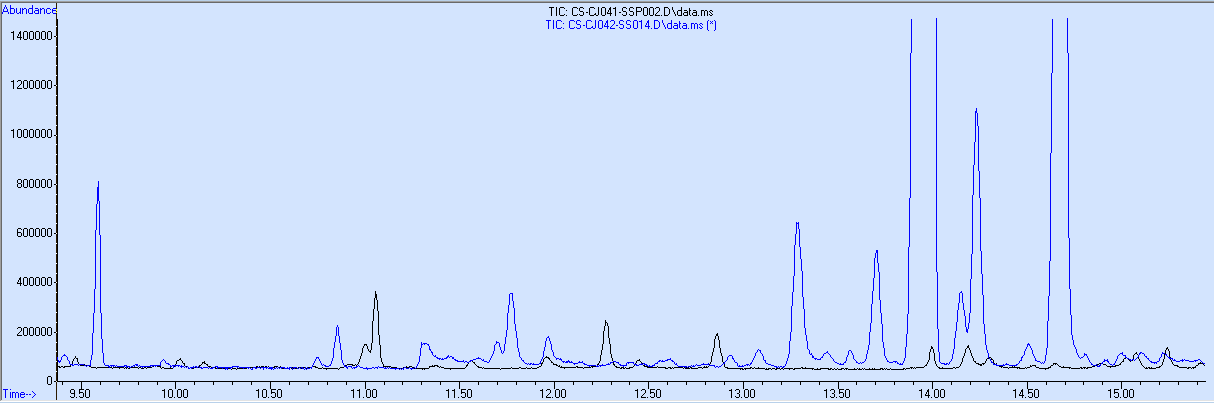


f

**Supplementary Figure S2**. Optimization of the sampling procedure.

Chromatograms of a Sorb-Star^®^ before **(a)** and after **(b)** the conditioning step.

Chromatogram overlays between 9.5 minutes and 16.5 minutes (where no silicone derivatives were observed) after dynamic sampling (in black) and static sampling (in blue) for 15 minutes **(c)**.

Chromatogram overlays after 5 minutes (in black), 10 minutes (in blue) and 15 minutes (in red) of dynamic sampling **(d)**.

Chromatogram of a Sorb-Star^®^ in contact for 15 minutes with a nitrile glove (in blue) and chromatogram of a Sorb-Star^®^ after the conditioning step (in dark) **(e)**.

Chromatogram overlays of a clean forehead sampling (in black) and the foaming gel (in blue) **(f)**.

| **Compound names** | **CAS number** | **Possible origins** |
| --- | --- | --- |
| **Acids** |  |  |
| Acetic acid | 64-19-7 | Cosmetics, Human metabolism |
| 2-Methyl-2-propenoic acid | 79-41-4 | Cosmetics |
| (E)-but-2-enoic acid (crotonic acid) | 3724-65-0 | Cosmetics |
| **Alcohols** |  |  |
| octan-1-ol | 111-87-5 | Human metabolism |
| ethane-1,2-diol | 107-21-1 | Human metabolism |
| decan-1-ol | 112-30-1 | Human metabolism |
| 1-(2-hydroxypropoxy)propan-2-ol | 110-98-5 | Human metabolism |
| dodecan-1-ol | 112-53-8 | Human metabolism |
| tridecan-1-ol | 112-70-9 | Human metabolism |
| 2-phenoxyethanol | 122-99-6 | Cosmetics |
| tetradecan-1-ol | 112-72-1 | Human metabolism |
| (3S)-butane-1,3-diol | 24621-61-2 | Cosmetics |
| undecan-1-ol | 112-42-5 | Cosmetics |
| pentadecan-1-ol | 629-76-5 | Cosmetics |
| octane-1,2-diol | 1117-86-8 | Cosmetics |
| menthol | 1490-04-6 | Cosmetics |
| 2,4,7,9-tetramethyldec-5-yne-4,7-diol | 126-86-3 | Cosmetics |
| 2-(2-hydroxyethoxy)ethanol (diethylene glycol) | 111-46-6 | Cosmetics |
| 2-(1-hydroxypropan-2-yloxy)propan-1-ol (dipropylene glycol) | 108-61-2 | Cosmetics |
| 2-butoxyethanol (ethylene glycol monobutyl ether) | 111-76-2 | Cosmetics |
| 2-(2-butoxyethoxy)ethanol (diethylene glycol monobutyl ether) | 112-34-5 | Cosmetics |
| 2-dodecoxyethanol (laureth-1) | 4536-30-5 | Cosmetics |
| 2-(2-ethoxyethoxy)ethanol (diethylene glycol monoethyl ether) | 111-90-0 | Cosmetics |
| (1S,5S)-2-methyl-5-propan-2-ylcyclohex-2-en-1-ol (cis-carvotanacetol) | 536-30-1 | Food |
| tetradecan-2-ol | 4706-81-4 | Food |
| dodecan-2-ol | 10203-28-8 | Cosmetics, Food |
| (3E)-4,8-dimethylnona-3,7-dien-2-ol | 67845-50-5 | Food |
| 1-(2-butoxyethoxy)ethanol | 54446-78-5 | Cosmetics |
| 2-ethyl-2-methyltridecan-1-ol | 921600-09-1 |  |
| (2,5-dimethyl-3,4-dihydropyran-2-yl)methanol | 54004-34-1 |  |
| 3,9-diethyltridecan-6-ol | 123-24-0 |  |
| **Aldehydes** |  |  |
| dodecanal | 112-54-9 | Human metabolism |
| 3-methylbut-2-enal | 107-86-8 | Food, Human metabolism |
| heptanal | 111-71-7 | Human metabolism |
| octanal | 124-13-0 | Food, Human metabolism |
| nonanal | 124-19-6 | Food, Human metabolism |
| decanal | 112-31-2 | Food, Human metabolism |
| (2E)-3,7-dimethylocta-2,6-dienal (citral) | 5392-40-5 | Cosmetics, Food |
| (E)-2-methylpent-2-enal | 623-36-9 | Food |
| 2,6-dimethylhept-5-enal | 106-72-9 | Cosmetics |
| **Alkanes** |  |  |
| hexadec-1-ene | 629-73-2 | Cosmetics |
| undecane | 1120-21-4 | Human metobolism |
| 2-methylhexadecane | 1560-92-5 | Food |
| tridecane | 629-50-5 | Cosmetics |
| octadecane | 593-45-3 | Cosmetics |
| heptadecane | 629-78-7 | Cosmetics |
| nonadecane | 629-92-5 | Cosmetics |
| heneicosane | 629-94-7 | Cosmetics |
| docosane | 629-97-0 | Cosmetics |
| tetradecane | 629-59-4 | Cosmetics |
| (E)-dodec-3-ene | 7206-14-6 | Human metabolism |
| dodec-1-ene | 112-41-4 | Cosmetics |
| hexadecane | 544-76-3 | Human metabolism |
| pentadecane | 629-62-9 | Human metabolism |
| 2,6,10,14-tetramethylhexadecane (phytane) | 638-36-8 | Food |
| dodecane | 112-40-3 | Cosmetics |
| decane | 124-18-5 | Cosmetics |
| eicosane | 112-95-8 | Cosmetics |
| 3-methylhexadecane | 6418-43-5 | Human metabolism |
| 8-methylheptadecane | 13287-23-5 | Human metabolism |
| (6E)-2,6-dimethylocta-2,6-diene | 2609-23-6 | Human metabolism |
| tetradec-3-ene | 41446-67-7 | Food |
| 3-methyltridecane | 6418-41-3 | Food |
| 2-methylpentadecane | 1560-93-6 | Food, Human metabolism |
| 4-methylpentadecane | 2801-87-8 | Food, Human metabolism |
| squalene | 111-02-4 | Human metabolism |
| 2,6,11-trimethyldodecane | 31295-56-4 | Food |
| (Z)-β-farnesene | 28973-97-9 | Cosmetics |
| 2,2,4,4,6,8,8-heptamethylnonane | 4390-04-9 | Cosmetics |
| tetradec-1-ene | 1120-36-1 | Cosmetics |
| 2,6,10-dimethyldodecane | 3891-98-3 | Cosmetics |
| 2,6,10,14-tetramethylpentadecane (pristane) | 1921-70-6 | Cosmetics |
| (3E,5E)-2,6-dimethylocta-1,3,5,7-tetraene | 460-01-5 | Food |
| tetradec-7-ene | 41446-63-3 | Human metabolism |
| (6E,10E)-7,11,15-trimethyl-3-methylidenehexadeca-1,6,10,14-tetraene | 70901-63-2 | Food |
| 4-methylheptadecane | 26429-11-8 | Food |
| 7-methylheptadecane | 20959-33-5 | Human metabolism |
| 7-methylpentadecane | 6165-40-8 | Human metabolism |
| 6-methylpentadecane | 10105-38-1 | Human metabolism |
| 5-methylpentadecane | 25117-33-3 | Human metabolism |
| 1-tert-butyl-2-methylbenzene | 1074-92-6 | Cosmetics |
| 4-methyltetradecane | 25117-24-2 | Food |
| 4-methylhexadecane | 25117-26-4 | Food, Human metabolism |
| 4,6-dimethyldodecane | 61141-72-8 | Food |
| 3-methyltetradecane | 18435-22-8 | Food |
| 3-methylpentadecane | 2882-96-4 | Food, Human metabolism |
| 3-methylheptadecane | 6418-44-6 | Food |
| 2,6,10-trimethyltetradecane | 14905-56-7 | Food |
| (Z)-dodec-6-ene | 7206-29-3 | Human metabolism |
| 2-methyltetradecane | 1560-95-8 |  |
| 2,6,10-trimethylpentadecane | 3892-00-0 |  |
| 2,2,6,6-tetramethyl-4-methyleneheptane | 141-70-8 |  |
| 2,4,4,6,6,8,8-heptamethylnon-1-ene | 15796-04-0 |  |
| 2-azido-2,4,4,6,6,8,8-heptamethylnonane | - |  |
| 5,8-diethyldodecane | 24251-86-3 |  |
| 7-methylhexadecane | 26730-20-1 |  |
| 8-hexylpentadecane | 13475-75-7 |  |
| 6-methyloctadecane | 10544-96-4 |  |
| 4-methyloctadecane | 10544-95-3 |  |
| 9-methylnonadecane | 13287-24-6 |  |
| 4-methylnonadecane | 25117-27-5 |  |
| **Cyclic Alkanes** |  |  |
| 3,7,7-trimethylbicyclo[4.1.0]hept-3-ene (3-carene) | 13466-78-9 | Cosmetics, Food |
| limonene | 138-86-3 | Cosmetics, Food |
| decan-5-ylbenzene | 4537-11-5 | Environment, Food |
| undecan-6-ylbenzene | 4537-14-8 | Environment, Food |
| dodecan-4-ylbenzene | 2719-64-4 | Environment, Food |
| undecan-2-ylbenzene | 4536-88-3 | Environment, Food |
| β-pinene | 127-91-3 | Cosmetics |
| longifolene | 475-20-7 | Cosmetics |
| undecan-5-ylbenzene | 4537-15-9 | Environment |
| undecan-4-ylbenzene | 4536-86-1 | Environment |
| undecan-3-ylbenzene | 4536-87-2 | Environment |
| dodecan-6-ylbenzene | 2719-62-2 | Environment |
| undecan-2-ylbenzene | 4536-88-3 | Environment |
| tridecan-6-ylbenzene | 4534-49-0 | Environment |
| dodecan-2-ylbenzene | 2719-61-1 | Environment |
| tridecan-4-ylbenzene | 4534-51-4 | Environment |
| tridecan-3-ylbenzene | 4534-52-5 | Environment |
| p-xylene | 106-42-3 | Environment |
| isolongifolene | 1135-66-6 | Cosmetics |
| β-iso-methyl ionone | 79-89-0 | Cosmetics |
| α-phellandrene | 99-83-2 | Cosmetics |
| sylvestrene | 1461-27-4 | Food |
| 2-ethenyl-1,1-dimethyl-3-methylidenecyclohexane | 95452-08-7 | Food |
| (+)-carvomenthene | 1195-31-9 | Food |
| tridecan-5-ylbenzene | 4534-50-3 | Environment, Food |
| longicyclene | 1137-12-8 | Cosmetics |
| α-Curcumene | 1461-02-5 | Food |
| γ-pyronene | 514-95-4 | Human metabolism |
| decan-2-ylbenzene | 4537-13-7 |  |
| tricyclo[10.2.1.02,11]pentadeca-4,8-diene | 74708-73-9 |  |
| 1,2,5,5-tetramethylcyclopenta-1,3-diene | 4249-12-1 |  |
| 1,2-dimethyl-3-pentyl-4-propylcyclohexane | 62376-17-4 |  |
| undecan-2-ylcyclohexane | 13151-77-4 |  |
| dodecan-5-ylbenzene | 2719-63-3 |  |
| **Esters** |  |  |
| (4-tert-butylcyclohexyl) acetate | 32210-23-4 | Cosmetics |
| hexyl 2-hydroxybenzoate | 6259-76-3 | Cosmetics |
| propyl hexadecanoate | 2239-78-3 | Cosmetics |
| ethyl myristate | 124-06-1 | Cosmetics |
| isopropyl myristate | 110-27-0 | Cosmetics |
| lauryl acrylate | 2156-97-0 | Cosmetics |
| dibutyl adipate | 105-99-7 | Cosmetics |
| isobornyl acetate | 125-12-2 | Cosmetics |
| dibutyl maleate | 105-76-0 | Cosmetics |
| diisobutyl maleate | 14234-82-3 | Cosmetics |
| isoamyl laurate | 6309-51-9 | Cosmetics |
| octyl octanoate | 2306-88-9 | Cosmetics |
| 2-hydroxyethyl acetate (ethylene glycol monoacetate) | 542-59-6 | Cosmetics |
| 2-hydroxyethyl propanoate (ethylene glycol monopropionate) | 24567-27-9 | Cosmetics |
| 2,2,4-trimethyl-3-carboxyisopropyl, isobutyl pentanoate | - | Food |
| 2-methoxyethyl tridecanoate | - | Environment |
| (3-hydroxy-2,3-dihydro-1H-inden-1-yl) acetate | - | Food |
| 1-[2-(isobutyryloxy)-1-methylethyl)]-2,2-dimethylpropyl 2-methylpropanoate | - | Food |
| [(1S,2R)-2-methylcyclopentyl] acetate | 40991-93-3 |  |
| pentadecan-4-yl 2-phenylacetate | - |  |
| (2-ethyl-3-hydroxyhexyl) 2-methylpropanoate | 74367-31-0 |  |
| 3-methylbutyl oct-2-ynoate | 68555-60-2 |  |
| 6-ethyloctan-3-yl octanoate | - |  |
| 1-octoxyoctane | 629-82-3 | Cosmetics |
| 8-propoxycedrane | 19870-75-8 | Food |
| 1-methoxyoctane | 929-56-6 | Environment |
| **Halogen compounds** |  |  |
| 1-chlorododecane | 112-52-7 | Environment |
| 1-chlorotetradecane | 2425-54-9 | Environment |
| (1E,5E)-1,6-dichlorocycloocta-1,5-diene | 29480-42-0 | Environment |
| 2-chlorobuta-1,3-diene | 14523-89-8 | Environment |
| 1-chlorohexadecane | 4860-03-1 | Environment |
| 1-(chloromethyl)-4-methylbenzene | 104-82-5 | Environment |
| 1-chloro-5-(1-chloroethenyl)cyclohexene | 13547-07-4 | Environment |
| 1-chloro-4-(1-chloroethenyl)cyclohexene | 13547-06-3 | Environment |
| **Heterocyclic compounds** |  |  |
| 2-pentylfurane | 3777-69-3 | Cosmetics |
| (2R,5S)-2-Methyl-5-(prop-1-en-2-yl)-2-vinyltetrahydrofuran | 54750-69-5 | Food |
| **Ketones** |  |  |
| geranyl acetone | 689-67-8 | Cosmetics, Food, Human metabolism |
| 6-methylhept-5-en-2-one | 110-93-0 | Human metabolism |
| tridecan-2-one | 593-08-8 | Cosmetics |
| hexadecan-2-one | 18787-63-8 | Food, Human metabolism |
| pentadecan-2-one | 2345-28-0 | Food |
| heptan-2-one | 110-43-0 | Cosmetics |
| menthone | 10458-14-7 | Cosmetics |
| β-methylionone | 127-43-5 | Cosmetics |
| 1-hydroxypropan-2-one | 116-09-6 | Food, Human metabolism |
| 2-(1-nitropropan-2-yl)cyclohexan-1-one | - | Food |
| heptadecan-2-one | 2922-51-2 | Food |
| 1-(cyclohexen-1-yl)ethanone | 932-66-1 |  |
| 3,6-dimethyloctan-2-one | 118452-32-7 |  |
| **Nitrogen compounds** |  |  |
| acetamide | 60-35-5 | Environment |
| N,N-dimethyldodecylamine | 112-18-5 | Cosmetics |
| N,N-dimethylformamide | 68-12-2 | Environment |
| acrylonitrile | 107-13-1 | Environment |
| aniline | 62-53-3 | Environment |
| N,N-dibutylformamide | 761-65-9 | Environment |
| N,N-dibutylacetamide | 1563-90-2 | Environment |
| methoxy-phenyl-oxime | 67160-14-9 | Environment |
| dibutylamine | 111-92-2 | Environment |
| cyclohex-3-ene-1-carbonitrile | 100-45-8 |  |
| 1-pentylpyrrole | 699-22-9 |  |
| 3-(dimethylamino)propanenitrile | 1738-25-6 |  |
| **Phenols** |  |  |
| butylated hydroxytoluene (BHT) | 128-37-0 | Cosmetics |
| 1,2-diethyl-4-phenylbenzene | 61141-66-0 |  |

**Supplementary Table S2.** Volatile organic compounds identified on the forehead before and after stress induction, classified by chemical class (in bold letters, alphabetically). Each compound was linked to a CAS number, and a possible origin was proposed.
